# Origins of cancer: tackling provocative questions

**DOI:** 10.18632/genesandcancer.150

**Published:** 2017-07

**Authors:** Abigail R. Solitro, Nicole A. Vander Schaaf

**Affiliations:** ^1^ Center for Cancer and Cell Biology, Van Andel Research Institute, Grand Rapids, MI, USA; ^2^ Center for Epigenetics, Van Andel Research Institute, Grand Rapids, MI, USA

**Keywords:** cancer, provocative questions initiative, microbiome, immunotherapy, biomarkers

## Abstract

Despite the tremendous progress that scientists have made throughout the history of cancer research, there are still far too many deaths and remaining scientific questions for us to be content with our current knowledge of the disease. The eighth Origins of Cancer symposium, held July 21, 2017 at Van Andel Research Institute, was organized around the theme of “Tackling Provocative Questions” to stimulate discussion of several of these unresolved paradoxes in the field of cancer research. The symposium highlighted recent progress from the National Cancer Institute's Provocative Questions Initiative, a program that offers research support to scientists who propose innovative strategies to address one of the featured questions. Accordingly, each of our eight distinguished speakers had received funding through this Initiative or performs research that closely aligns with one of these important yet understudied questions. From microbes to biomarkers to immunotherapy, this meeting report describes the latest advancements that were presented at the symposium.

## INTRODUCTION

The 2017 Origins of Cancer symposium pushed the boundaries of understudied and overlooked aspects of cancer research. The symposium boasted eight renowned clinicians and scientists whose research is funded by or closely aligns with the mission of the National Cancer Institute's (NCI's) Provocative Questions Initiative. Established by former National Institutes of Health (NIH) director, Dr. Harold Varmus, the Provocative Questions Initiative has sought to shed light on controversial, unanswered, and truly provocative questions in the cancer field since 2011 [1]. This year's symposium highlighted recent findings related to five of the provocative questions: (1) “what are the underlying molecular mechanisms that are responsible for the functional differences between benign proliferative diseases and premalignant states?”; (2) “how do microbiota affect the response to cancer therapies?”; (3) “how do variations in tumor-associated immune responses contribute to differences in cancer risk, incidence, or progression?”; (4) “what cancer models or other approaches can be developed to study clinically stable disease and the subsequent transition to progressive disease?”; and (5) “can we develop bifunctional small molecules that will couple oncoproteins or other cancer-causing molecules of interest to inactivating processes such as degradation and achieve tissue-specific loss of function?”

The symposium began with an introduction and overview of the Provocative Questions Initiative by NCI's Program Director for the Initiative, Dr. Emily Greenspan. Dr. Greenspan shared that since 2011, the Initiative has received over 2,000 grant applications resulting in over 200 new R01 and R21 awards, totaling over $200 million in research support. This success is attributed to the iterative cycle of question generation, evaluation, and revision, a process that includes both scientists and clinicians from multiple disciplines; frequent workshops are held around the world, hosting brainstorming sessions for experts in the cancer research field to debate the most important questions to be answered. Although the Initiative is still in its early years, recent evaluations of its success are promising. Dr. Greenspan shared that 30 of the 33 questions issued thus far have represented less than one percent of cancer literature, suggesting that these areas truly are understudied. In addition, over half of the questions’ research areas saw an increase in cancer literature since the inception of the program. On average, each award funded by the Initiative has produced four publications, and these publications are cited twice as frequently as those not funded by the Initiative. Clearly, the NCI's support of innovative and creative research promises to benefit the cancer research community and ultimately, the individuals affected by these diseases.

### What are the underlying molecular mechanisms that are responsible for the functional differences between benign proliferative diseases and premalignant states?

Following the introduction of the Initiative, the first provocative question that was addressed was, “what are the underlying molecular mechanisms that are responsible for the functional differences between benign proliferative diseases and premalignant states?” This question addresses the critical need for accurately diagnosing disease in order to prevent unnecessary or mistreatment of patients. Dr. Chetan Bettegowda (Johns Hopkins University School of Medicine) discussed his research using neurofibromatosis (NF) as a model of a benign proliferative disease that can advance to malignancy in the form of malignant peripheral nerve sheath tumors (MPNSTs). Dr. Bettegowda has shown that distinct, sequential mutations cause the progression of this disease; germline and subsequent somatic mutations in the *NF1* gene lead to benign NF, and mutations in polycomb repressive complex 2 (PRC2) cause MPNSTs to arise from plexiform disease. Recently, Dr. Bettegowda's group uncovered the importance of *SUZ12* mutations in this progression [2]. Interestingly, *SUZ12* sits next to the *NF1* gene on chromosome 17, and both are frequently lost in large deletion events. Dr. Bettegowda hopes to utilize these disease-associated mutations as biomarkers in NF patients. Using colorectal cancer (CRC) as a model, his team has shown that circulating tumor DNA can be collected, amplified, and used to determine the mutation status of the parent tumor [3, 4]. Current research continues to apply this technique to patients with MPNSTs to inform treatment decisions.

Building off of Dr. Bettegowda's recent advances, Dr. Lisa Boardman (Mayo Clinic College of Medicine) discussed her research utilizing the progression of colon polyps to CRC as a model for addressing this provocative question. Dr. Boardman shared that the current standard for CRC screening is the colonoscopy, of which over six million are performed in the United States annually. She elaborated that about 25-40% of these procedures reveal polyps, but only about 10% of these polyps will progress to cancer. Unfortunately, the distinguishing features between polyps that progress to malignancy and those that remain benign are unknown. Dr. Boardman's group seeks to fill this gap in knowledge. Recently, Dr. Boardman has collected tissue samples from CRC tumors, cancer-adjacent polyps, and cancer-free polyps in order to perform exploratory data analysis at the DNA, RNA, and epigenetic levels [5, 6]. Several key findings have been elucidated from this work; while *KRAS* and *APC* mutations are common among all three tissue groups, *TP53* mutations are only present in tumors and cancer-adjacent polyps. In addition, global gene expression signatures distinguish tumors and cancer-adjacent polyps from cancer-free polyps, with *CXCL5* expression elevated in the former, for example. Using reduced representation bisulfite sequencing, she also found that tumors and cancer-adjacent polyps tend to have more DNA methylation than cancer-free polyps. Dr. Boardman plans to continue investigating patient samples using various sequencing technologies, with the hope of integrating these data to create a panel of markers that will identify all patients whose polyps have the potential to progress to CRC.

### How do microbiota affect the response to cancer therapies?

In the second session, Drs. Scott Bultman (University of North Carolina at Chapel Hill) and Chrystal Paulos (Medical University of South Carolina) discussed the provocative question, “how do microbiota affect the response to cancer therapies?” The purpose of this question is to better understand the role of the microbiome in cancer, with the long-term goal of developing adjuvant therapies to enhance cancer treatment by targeting the microbiota. Dr. Bultman's talk focused on the microbial byproduct butyrate, a short-chain fatty acid and naturally occurring histone deacetylase (HDAC) inhibitor [7]. Butyrate is a bacterial fermentation product of fiber and is prevalent in the colon. Using the azoxymethane (AOM) and dextran sodium sulfate (DSS) mouse model of CRC, he found that colonizing the mice with butyrate-producing *Butyrivibrio fibrisolvens* reduces the tumor burden in mice on a high-fiber diet [8]. Neither a high-fiber diet alone nor colonization by a *B. fibrisolvens* mutant that cannot produce butyrate provides this tumor-suppressive effect. Interestingly, the impact of butyrate depends on the oxidative state of the target cell; normal colonic cells use butyrate as their primary energy source, so butyrate enhances their proliferation. In contrast, butyrate slows the proliferation of CRC cells, likely due to their shifted dependence on glucose because of the Warburg effect and consequent accumulation of the natural HDAC inhibitor [9]. These studies highlight the important role that microbial metabolites play in altering host physiology in both health and disease.

Dr. Chrystal Paulos presented her discovery that microbiota may explain how lymphodepletion enhances adoptive T cell transfer (ACT) therapy. For ACT therapy, T cells are removed from a patient and co-cultured *ex vivo* with the patient's own cancer cells. Cancer-reactive T cells are then selected, expanded, and returned to the patient, hopefully inducing a robust immune response against the cancer cells. When coupled with lymphodepletion (irradiation of the patient's immune cells), the efficacy of ACT therapy is enhanced, but the reasons for this are not well understood [10-13]. Dr. Paulos finds that treatment with the antibiotic Ciprofloxacin diminishes the benefit of lymphodepletion, suggesting that bacteria are partially responsible [14]. Her current working model is that lymphodepletion enhances ACT therapy through microbe-driven activation of the innate immune system. The radiation that is used for lymphodepletion damages the intestinal epithelial barrier and allows bacteria to penetrate regions where they are normally not permitted. This translocation activates innate immune cells, which foster the survival and activation of the re-introduced cancer-reactive T cells. Dr. Paulos hopes that these studies will reveal novel strategies to safely and effectively augment cancer immunotherapy.

### How do variations in tumor-associated immune responses contribute to differences in cancer risk, incidence, or progression?

In the third session, Drs. Timothy Chan (Memorial Sloan Kettering Cancer Center) and Lyse A. Norian (University of Alabama at Birmingham) addressed the following question: “how do variations in tumor-associated immune responses contribute to differences in cancer risk, incidence, or progression?” This provocative question explores the factors that contribute to differential tumor-associated immune responses between patients, with the long-term goal of developing strategies to enable a patient's immune system to prevent or eradicate cancer. Dr. Chan uses computational strategies to identify molecular features that may help us understand why some patients respond to immunotherapy while others do not. He highlighted the importance of neoantigens (peptides that have undergone mutations that make them appear as foreign to the immune system) for achieving robust, cancer-targeted immune responses. Indeed, he found that mutational burden is predictive of response to anti-PD1 therapy in non-small cell lung cancer patients, with responsive patients having a higher average nonsynonymous mutation load than non-responders [15]. This trend holds true in several other cancers and may be a valuable tool for deciding a patient's treatment regimen. Recent studies, however, indicate that not all neoantigens are equal; for example, clonal neoantigens and possibly those resembling epitopes of pathogens appear to be more immunogenic [16-19]. Future studies will continue to explore how the mutational landscape and burden as well as neoantigen sequence and clonality contribute to the differential tumor-associated immune responses between patients.

Dr. Norian studies obesity as a risk factor for cancer and the impact that obesity has on immune responses in health and disease. Obesity increases the risk of 13 different types of cancer, but the precise mechanism is unknown [20]. Through her studies in mice with renal tumors, Dr. Norian finds that mice with diet-induced obesity respond poorly to cancer immunotherapy as compared to mice on a normal diet. Despite this phenotype, obesity does not significantly impact the development or maintenance of pre-existing or newly stimulated CD8 T cell responses [21]. However, Dr. Norian discovered that renal tumors from obese mice have enhanced accumulation of myeloid-derived suppressor cells, which are known to be immunosuppressive, and this recruitment is likely due to a higher intratumoral concentration of the myeloid cell-attractant CCL2 [22]. In this environment, dendritic cells have a reduced ability to activate T cells, which may help explain why obese mice have dampened responses to cancer immunotherapy. Dr. Norian hopes that these findings will enable scientists to develop dietary regimens or drugs that will enhance immune surveillance and responses to immunotherapy in cancer patients.

### What cancer models or other approaches can be developed to study clinically stable disease and the subsequent transition to progressive disease?

The next provocative question that was addressed was, “what cancer models or other approaches can be developed to study clinically stable disease and the subsequent transition to progressive disease?” This question encourages the use of creative and non-traditional models that more closely mimic human disease. Dr. Christine Iacobuzio-Donahue (Memorial Sloan Kettering Cancer Center) utilizes autopsies as a means of modeling pancreatic cancer progression. Primary tumors, metastases and normal tissues such as blood, and cerebrospinal fluid are removed from deceased patients; tumors and metastases are then used to establish patient-derived cell lines and xenograft mouse models. Her recent work has uncovered an incredible degree of mutational heterogeneity between different regions of the same tumor and/or metastasis [23]. At the same time, mutations in the four canonical pancreatic cancer-associated genes (*KRAS*, *TP53*, *CDKN2A*, and *SMAD4*) were consistently found in all tumors and metastases [24]. These unique mutational signatures have shown that local, recurring tumors are more similar to metastases than to the primary tumor, suggesting that metastases have the ability to seed tumor cells back into the pancreas after primary tumor resection. Dr. Iacobuzio-Donahue hopes to continue elucidating the importance of heterogeneity in pancreatic cancer in order to exploit it for therapeutic benefit to patients.

### Can we develop bifunctional small molecules that will couple oncoproteins or other cancer-causing molecules of interest to inactivating processes such as degradation and achieve tissue-specific loss of function?

The final speaker of this year's symposium was Dr. Craig Crews (Yale University) who shared his insight into the provocative question, “can we develop bifunctional small molecules that will couple oncoproteins or other cancer-causing molecules of interest to inactivating processes such as degradation and achieve tissue-specific loss of function?” The Crews lab has developed a “chemical equivalent of CRISPR” called PROTACs (proteolysis-targeting chimeras), small molecules that fuse ligands for a target protein to E3 ubiquitin ligase recognition domains [25]. The theory behind PROTACs is that oncogenic proteins or other cancer-causing molecules can be selectively targeted for degradation by the cell's own proteasome system. Dr. Crews has shown the success of PROTAC technology using the von Hippel-Lindau E3 ubiquitin ligase recognition domain and several target proteins, including RIPK and ERRα [26, 27]. Of clinical importance, his recent work in mouse models suggests that PROTACs are orally available, tolerated, and effective [28]. Dr. Crews hopes to continue developing this technology for application to undruggable targets such as KRAS. This approach has the potential to revolutionize cancer therapy and is an exemplary illustration of using innovation to accomplish what was formerly deemed impossible.

The 2017 Origins of Cancer symposium successfully met its aim of providing a platform for discussion of unanswered and important questions in cancer research. From accurately diagnosing benign and malignant diseases to exploring the role of the immune system in cancer, these provocative topics are at the cutting edge of advancing our understanding of these diseases. The studies being performed by these eight scientists and their teams promise to impact not only their respective fields but also clinical care. We were honored to host them at this annual symposium and look forward to the stimulating discussion that awaits us at the next Origins of Cancer symposium, to be held at Van Andel Research Institute in July of 2018.
